# Associations between Insomnia Symptoms and Anxiety Symptoms in Adults in a Community Sample of Southeastern Pennsylvania, USA

**DOI:** 10.3390/diseases10040092

**Published:** 2022-10-18

**Authors:** Sadia B. Ghani, Ashna Kapoor, Andrew S. Tubbs, Chloe C. A. Wills, Jordan F. Karp, Michael L. Perlis, William D. S. Killgore, Fabian-Xosé Fernandez, Michael A. Grandner

**Affiliations:** 1 Sleep and Health Research Program, Department of Psychiatry, University of Arizona College of Medicine, Tucson, AZ 85724, USA; 2 Department of Psychiatry, University of Arizona College of Medicine, Tucson, AZ 85724, USA; 3 Perelman School of Medicine, University of Pennsylvania, Philadelphia, PA 19104, USA; 4 SCAN Lab, Department of Psychiatry, University of Arizona College of Medicine, Tucson, AZ 85724, USA; 5 Department of Psychology, University of Arizona, Tucson, AZ 85724, USA

**Keywords:** insomnia, anxiety, daytime dysfunction, sleep health, short sleep

## Abstract

Although insomnia is reliably associated with anxiety symptoms, aspects of insomnia may differentially relate to one anxiety symptom versus another. Therefore, treatment for insomnia comorbidity with anxiety might be individually tailored to optimize treatment response. Working from this hypothesis, we analyzed data from a survey of 1007 community-dwelling adults. Insomnia was measured using the Insomnia Severity Index (ISI), categorizing items as nighttime disturbances, daytime dysfunction, or self-perceived dissatisfaction. Anxiety symptoms were measured with the Generalized Anxiety Disorder 7-item questionnaire (GAD-7). Linear and binomial logistic regression were used and adjusted for covariates. Post hoc forward stepwise analyses determined which components of the insomnia contributed to individual anxiety symptoms. Significant associations between nighttime disturbance (β = 0.88 [0.44, 1.3]), daytime dysfunction (β = 1.30 [0.81, 1.80]), dissatisfaction (β = 1.20 [0.60, 1.7]) and total GAD-7 score were maintained after adjusting for covariates. Nighttime disturbance was associated with excess worrying, restlessness, irritability, and fear of catastrophe. Daytime dysfunction was associated with all symptoms except for fear of catastrophe, and self-perceived dissatisfaction was associated with all symptoms except irritability. Stepwise analyses revealed that daytime dysfunction and dissatisfaction were most consistently related to anxiety symptoms. Greater attention should be paid to daytime dysfunction in patients with insomnia and anxiety, as improving daytime functioning may improve anxiety.

## 1. Introduction

There is strong and consistent evidence of a bidirectional relationship between anxiety disorders and insomnia [1,2,3,4,5]. Complaints of insomnia often precede, accompany, and/or follow the incidence or exacerbation of anxiety symptoms, and anxiety is among the most common comorbid conditions in individuals with insomnia [6]. Indeed, in a population-based sample, nearly half of the adult patients with insomnia report significant anxiety symptoms, and 13% meet the criteria for generalized anxiety disorder (GAD) [7]. The close relationship between these two disorders is also reflected in the Diagnostic and Statistical Manual of Mental Disorders—Fifth Edition (DSM-V) diagnostic criteria for GAD [8], where disturbances of sleep continuity constitute one of the six possible symptoms. Consequently, ongoing research has identified genetic, biological, and even environmental factors that may link insomnia with anxiety and other psychiatric illnesses [9,10,11,12,13,14]. 

Insomnia, however, is not a unitary construct, nor does it only manifest as a disruption in sleep continuity at night. Factor analyses of the Insomnia Severity Index (ISI) [15] typically identify at least two distinct components [16,17,18,19]: nighttime disturbance (disturbances of sleep continuity) and daytime dysfunction. An additional component, self-perceived dissatisfaction, has also been noted [16,17]. The distinctions matter because different manifestations of insomnia have disparate impacts; a study of Swedish workers, for instance, found that sleep onset insomnia was a stronger predictor of receiving disability than other nighttime disturbances [20]. The relationship between insomnia and anxiety is further complicated by sleep duration, as insomnia with short sleep is linked to greater neurocognitive impairment, more psychological distress, and ultimately more severe disease [21,22,23,24]. These findings raise the question of whether specific features of insomnia, in conjunction with sleep duration, have distinct implications for the severity or presentation of comorbid anxiety, as sleep continuity disturbances contribute to rising anxiety levels, and different combinations of insomnia symptoms correspond with different strengths of anxiety symptoms [25]. Therefore, it is not unreasonable to think that some insomnia sub-constructs are more indicative of severity of illness and may be a useful marker for possible comorbidities (i.e., anxiety or depression). This is of clinical importance, as associations between insomnia constructs and specific anxiety symptoms may help guide health professionals toward more individualized treatments.

To address these questions, the present study explored how the three components of insomnia (nighttime disturbance, daytime dysfunction, and self-perceived dissatisfaction) were related to individual symptoms of anxiety among community-dwelling adults. The a priori hypothesis was that nighttime disturbance would be most strongly associated with overall anxiety severity and with greater reporting of individual anxiety symptoms because of the resulting deficits in sleep compared to daytime dysfunction and self-perceived dissatisfaction. A secondary analysis examined whether subjective sleep duration moderated any of these associations. 

## 2. Materials and Methods

### 2.1. Data Source

Data were derived from the Sleep and Healthy Activity, Diet, Environment and Socialization (SHADES) study, an internet-based questionnaire study of *n* = 1007 adults aged 22–60, residing in the greater Philadelphia area. Participants were recruited through advertisements and outreach to local community centers. The study included completion of a survey either online or in-person, consisting of questions regarding sleep habits, health, behavior, and environmental factors. Respondents were required to answer all questions to receive compensation, and only completed surveys were used for analysis. The Institutional Review Board from the University of Pennsylvania approved this study, and informed consent was obtained from all participants and in accordance with the Code of Ethics of the World Medical Association (Declaration of Helsinki).

### 2.2. Measures

Insomnia in the past 2 weeks was measured using the Insomnia Severity Index (ISI) [15], which assesses seven symptoms using a 0 to 4 Likert scale. Items 1, 2, and 3 measure difficulty falling asleep, staying asleep, or waking too early (nighttime disturbances), while items 5 and 6 measure the impact on daytime functioning and quality of life (daytime dysfunction). Items 4 and 7 measure worry/distress about sleep and dissatisfaction with the current sleep pattern (self-perceived dissatisfaction). These three insomnia components have been previously validated in this sample [16], and so, each component was calculated by summing the relevant items. The summed scores between 0 and 7 were indicative of no insomnia, scores between 8 and 14 were indicative of subthreshold insomnia, scores between 15 and 21 were indicative of moderate insomnia and scores between 22 and 28 were indicative of severe insomnia. 

Anxiety was measured using the Generalized Anxiety Disorder 7-item questionnaire (GAD-7) [26], which assesses symptoms in the last 2 weeks on a 0 to 3 scale (0: “not at all”, 1: “several days”, 2: “more than half the days”, 3: “nearly every day”). The total GAD score was dichotomized as <10 points, or ≥10 points, where 10 points is considered a reasonable cut-off for identifying cases of moderate GAD [26]. The specific anxiety symptoms that were measured were: anxious feelings; inability to control worrying; excess worrying; trouble relaxing; restlessness; irritability; and catastrophizing. 

Sleep duration was assessed using a single question: “How much sleep do you usually get at night on weekdays or workdays?”. This question is derived from the National Health and Nutrition Examination Survey and has been used in prior studies [27]. Responses were provided as whole numbers and were categorized into “Short” (less than or equal to 6 h), “Recommended” (7 to 8 h), and “Long” (9 or more h) based on existing guidelines [28,29]. 

Self-reported age, sex, race/ethnicity (Non-Hispanic White, Black/African-American, Hispanic/Latino, Asian, and Other/Multiracial), level of education (college, some college, high school or less), income quintile, body mass index, and sleep apnea risk score were included as covariates. These were chosen because they reflect areas of overlap between sleep and mental health and because these were used in a previous study of depression symptoms [16]. Sleep apnea risk score was assessed using the multivariate apnea prediction index (MAP) [30]. This score is based on a prediction equation that includes snoring, age, sex, BMI, and self-reported symptoms of snorting or gasping, loud snoring and breathing difficulties during the night. These variables are entered into an equation producing an index score that ranges from 0 to 1, with 0 representing the lowest probability a patient has sleep apnea and 1 representing the highest probability a patient has sleep apnea. 

### 2.3. Data Analysis

Simple comparisons were made using Chi-squared tests. Linear and binomial logistic regression models estimated the associations between insomnia components and anxiety severity/symptoms. In Model 1, the three insomnia components were entered as the only predictors of total GAD score and the presence of individual anxiety symptoms. Model 2 was adjusted for all the study covariates. Model 3 followed Model 2 but examined only self-reported short sleepers. Finally, a post hoc forward stepwise regression analysis examined all three insomnia symptom categories as potential predictors and each individual anxiety symptom as outcome. Predictors were retained in the model if they explained significant otherwise unexplained variance (*p* = 0.05). In all models, the insomnia components were standardized to improve interpretability. Associations are reported as standardized regression coefficients (β) or standardized odds ratios (OR) with 95% confidence intervals (95% CI), with significance set at 0.05. All analyses were conducted in R (version 4.1.0, R Foundation for Statistical Computing, Vienna, Austria) and Stata (StataCorp LLC, College Station, TX, USA). 

## 3. Results

### 3.1. Characteristics of Sample

Table 1 presents the summary characteristics of the sample. The sample was majority female (61%), relatively young (mean age 34 ± 9.4), and majority non-Hispanic White (59.5%), although there was significant Black/African-American representation (25%). Most participants had a college degree (55.9%). The average ISI score was 10.6, with 268 individuals reporting clinically significant insomnia. The average GAD-7 score was 7.2, with 311 individuals reporting clinically significant anxiety (i.e., GAD ≥ 10). Those with clinically significant anxiety were more likely to exhibit short sleep, not get the recommended amount of daily sleep, and report moderate or severe insomnia (*p* < 0.05; Table 1).

As a secondary analysis, we explored associations between insomnia severity and demographic predictors among those who had higher levels of anxiety (GAD ≥ 10). The goal of this secondary analysis was to examine characteristics of those individuals who had higher anxiety but lower insomnia severity. In models restricted only to those with higher anxiety (*n* = 310), which included age, sex, education, and race/ethnicity, only education level was associated with insomnia severity. Compared to college graduates, those with some college were less likely to have mild (RRR = 0.55, 95% CI [0.31, 0.98], *p* = 0.041) or no (RRR = 0.32, 95% CI [0.13, 0.80], *p* = 0.014) insomnia symptoms, those with only a high school education were less likely to have mild (RRR = 0.022, 95% CI [0.09, 0.54], *p* = 0.001) or no (RRR = 0.12, 95% CI [0.02, 0.59], *p* = 0.010) insomnia symptoms, and those with less than a high school education were less likely to have mild (RRR = 0.17, 95% CI [0.03, 0.86], *p* = 0.032) insomnia symptoms.

### 3.2. The Relationship between Insomnia Components and Severity of Anxiety

In Model 1, all three insomnia components were independently and significantly associated with total GAD-7 score (Nighttime Disturbance: β = 0.88 [0.44, 1.3]; Daytime Dysfunction: β = 1.3 [0.81, 1.8]; Self-Perceived Dissatisfaction: β = 1.2 [0.60, 1.7], all *p* < 0.001). Adjusting for sociodemographic covariates did not significantly modify these results, and there were no differences among only short sleepers. These results are presented in Table 2.

### 3.3. The Relationship between Insomnia Components and Individual Anxiety Symptoms

The associations between insomnia components and individual anxiety symptoms are presented in Table 3. In Model 1, nighttime disturbance was associated with excess worrying (OR: 1.23 [1.00, 1.51]), restlessness (OR: 1.57 [1.22, 2.03]), irritability (OR: 1.28 [1.04, 1.57]), and catastrophizing (OR: 1.31 [1.04, 1.65]). When adjusting for sociodemographic covariates (Model 2), nighttime disturbance was no longer associated with catastrophizing (*p* = 0.06) but was associated with inability to control worrying (OR: 1.26 [1.02, 1.56]). Among short sleepers (Model 3), however, nighttime disturbance was only associated with inability to control worrying (OR: 1.36 [1.02, 1.83]). 

Daytime dysfunction was significantly associated with six out of seven symptoms in Model 1: anxious feelings (OR: 1.73 [1.39, 2.16]), inability to control worrying (OR: 1.55 [1.23, 1.93]), excess worrying (OR: 1.62 [1.31, 2.03]), trouble relaxing (OR: 1.42 [1.13, 1.77]), restlessness (OR: 1.63 [1.25, 2.15]), and irritability (OR: 1.59 [1.27, 1.98]). Adjusting for sociodemographic covariates did not significantly modify these associations nor did these relationships differ among only short sleepers.

Finally, self-perceived dissatisfaction with sleep was associated with six out of seven anxiety symptoms in Model 1: anxious feelings (OR: 1.30 [1.01, 1.69]), inability to control worrying (OR: 1.54 [1.18, 2.00]), excess worrying (OR: 1.57 [1.22, 2.04]), trouble relaxing (OR: 1.89 [1.45, 2.47]), irritability (OR: 1.35 [1.04, 1.76]), and catastrophizing (OR: 1.52 [1.13, 2.05]). Adjusting for covariates did not significantly alter these relationships except for feeling anxious, which was no longer significant. Among short sleepers, self-perceived dissatisfaction was only associated with trouble relaxing (OR: 1.77 [1.24, 2.55]); all other relationships were not significant. 

### 3.4. Forward Stepwise Analysis between Insomnia Components and Individual Anxiety Symptoms

In the forward stepwise analysis (Table 4), daytime dysfunction was the primary component associated with anxious feelings, restlessness, and irritability, and was also associated with inability to control worrying, excess worrying, and difficulty relaxing. Self-perceived sleep dissatisfaction was the primary component associated with inability to control worrying, excess worrying, difficulty relaxing, and catastrophizing, and it was also associated with anxious feelings and irritability. While nighttime disturbance was associated with inability to control worrying, excess worrying, restlessness, and irritability, it was not the primary component for any one symptom.

## 4. Discussion

This study examined whether different components of insomnia were differentially associated with anxiety. In adjusted analyses, all three components were significantly associated with overall severity of anxiety, even among short sleepers. Contrary to the primary hypothesis, daytime disturbance and self-perceived dissatisfaction with sleep were each associated with more anxiety symptoms than nighttime disturbance. Moreover, in forward stepwise modeling, nighttime disturbance had the weakest associations with each symptom. These findings indicate that the nighttime features of insomnia may have less relevance to anxiety symptomatology than the ability to function properly during the day or subjective feelings of distress. 

The original hypothesis was that nighttime disturbance, the colloquial hallmark of insomnia, would be most strongly associated with overall anxiety severity and individual anxiety symptoms. However, nighttime disturbance generally had the weakest relationships; for overall anxiety severity, nighttime disturbance had the smallest beta coefficients and was significant among short sleepers. While this seems counterintuitive, prior research does indicate that nighttime disturbances are not the most salient feature of insomnia. In their analysis of sleep complaints, Koffel and Watson [19] described two components of sleep disturbance as either insomnia (nighttime symptoms) or lassitude (daytime symptoms), and they further reported that lassitude was more strongly associated with depression and anxiety than insomnia. Similarly, Ji and colleagues [16] reported that daytime dysfunction and self-perceived sleep dissatisfaction were associated with more depression symptoms than nighttime disturbances. Other studies assessed the relationship between insomnia and anxiety using different measurement tools. For instance, Bragantini and colleagues [25] in the HUNT3 study assessed insomnia symptoms using the DSM-5 [8] and anxiety using the Hospital Anxiety and Depression Scale, such that insomnia was categorized as sleep onset, sleep maintenance, or terminal insomnia. Despite the different scales, the HUNT3 study found that anxiety levels were higher in participants reporting symptoms of insomnia. 

As noted before, insomnia with objective short sleep may be the most severe phenotype among patients with insomnia [24]. Thus, patients with insomnia and short sleep may also display distinct relationships with anxiety symptoms. This certainly seemed true in the present study: nighttime disturbance and self-perceived dissatisfaction among short sleepers were each associated with one anxiety symptom, while daytime dysfunction remained associated with six symptoms. While hypothetical, an explanation may be differences in limbic activity, as emotion regulation has been shown to explain the relationship between anxiety and insomnia [31]. Thus, among patients with insomnia and short sleep, it is possible that emotional regulation may be so impaired that symptoms of anxiety are inevitable. Alternatively, another possible explanation is that neurocognitive deficits in insomnia with short sleep may obfuscate feelings of distress or even perceptions of sleep disturbances without impairing perceptions of daytime functioning. Of course, sleep duration was measured subjectively using a single, albeit widely used, item, so the accuracy of this measure is in question. Further research is needed to clarify how insomnia with short sleep is specifically linked to the severity and symptomatology of anxiety. 

These observations offer valuable insight into how patients with comorbid anxiety and insomnia should be treated. Perhaps instead of focusing solely on disturbed sleep, emphasizing daytime activity and structure for patients and methods to deal with subjective distress will better help these patients. Professionals could adjust a patient’s daytime routine, sleep cycle, dietary habit, and physical activity in accordance with the approaches provided by interpersonal and social rhythm therapy [32] and the “Trans-C” sleep and circadian rhythm intervention developed by Harvey and colleagues [33]. Additionally, an emphasis on daytime functioning may dissuade physicians from needlessly prescribing sedative–hypnotic medications, which is a common intervention that may worsen daytime sedation [34].

The strengths of this study are the large, community-based sample, including a large enough sub-sample of short sleepers to make the secondary analysis possible. Limitations of this analysis include the cross-sectional nature of the study, which does not allow for causal determinations. Thus, a replication of these findings using a longitudinally-followed cohort would advance the field. In addition, all data were self-reported, which means the accuracy of the insomnia data may be limited by recall and reporting biases. Prospective sleep diaries or objective measures of sleep would clarify this limitation. It should be noted that the “self-perceived dissatisfaction” symptoms of the ISI include items that may conceptually overlap with the concept of anxiety—specifically, these items represent worry and distress about sleep and dissatisfaction with sleep. Not surprisingly, the results of this study show that dissatisfaction/distress about sleep is related to general worry/distress associated with anxiety. This may represent a limitation of the ISI in studies of anxiety, since some of the items conceptually overlap. This study, though, allows for an exploration of aspects of the ISI that do not include worry or distress, showing that other aspects of insomnia, including the nighttime and daytime symptoms specifically, may have their own associations with anxiety. 

## 5. Conclusions

In analyzing data from over a thousand adults, the three components of insomnia (nighttime disturbance, daytime dysfunction, and self-perceived dissatisfaction) were significantly associated with overall anxiety severity. However, nighttime disturbance was only associated with four out of seven symptoms examined, compared to six out of seven for daytime dysfunction and self-perceived dissatisfaction. A restricted analysis of short sleepers found that daytime dysfunction remained associated with six out of seven symptoms, while nighttime disturbance and self-perceived dissatisfaction were associated with only one symptom each. It is well known that sleep continuity disturbances may contribute to daytime dysfunction, as daytime dysfunction may be a proxy of unmet sleep needs. However, the present data highlight that the daytime dysfunction and self-perceived dissatisfaction are important correlates of clinical anxiety. These findings emphasize the importance for health professionals in various specialties to address a combination of factors associated with both insomnia and anxiety. 

## Figures and Tables

**Table 1:** Characteristics of the sample by severity of anxiety.

Characteristic	Overall	GAD < 10	GAD ≥ 10	*p*
N	1007	696	311	
Age	34.0 (9.45)	34.0 (9.48)	33.9 (9.38)	0.800
BMI	26.6 (7.85)	26.3 (7.78)	27.5 (7.96)	0.024 *
**Sex**	**Overall**	**GAD < 10**	**GAD ≥ 10**	***p***
Male	388 (38.5%)	265 (38.1%)	123 (39.5%)	0.700
Female	619 (61.5%)	431 (61.9%)	188 (60.5%)	
**Race**	**Overall**	**GAD < 10**	**GAD ≥ 10**	***p***
Non-Hispanic White	597 (59.5%)	425 (61.3%)	172 (55.5%)	0.400
Black/African-American	251 (25.0%)	163 (23.5%)	88 (28.4%)	
Hispanic/Latino	46 (4.6%)	31 (4.5%)	15 (4.8%)	
Asian	54 (5.4%)	39 (5.6%)	15 (4.8%)	
Other/Multiracial	55 (5.5%)	35 (5.1%)	20 (6.5%)	
**Income Quintile**	**Overall**	**GAD < 10**	**GAD ≥ 10**	***p***
1 (lowest)	187 (18.6%)	137 (19.7%)	50 (16.1%)	0.1200
2	157 (15.6%)	113 (16.2%)	44 (14.1%)	
3	249 (24.7%)	178 (25.6%)	71 (22.8%)	
4	231 (22.9%)	154 (22.1%)	77 (24.8%)	
5 (highest)	183 (18.2%)	114 (16.4%)	69 (22.2%)	
**Education**	**Overall**	**GAD < 10**	**GAD ≥ 10**	***p***
College	563 (55.9%)	420 (60.3%)	143 (46.0%)	<0.001 *
Some college	312 (31.0%)	198 (28.4%)	114 (36.7%)	
High school or less	132 (13.1%)	78 (11.2%)	54 (17.4%)	
**Sleep Apnea Risk score**	**Overall**	**GAD <10**	**GAD ≥ 10**	***p***
MAP Score	−2.0 (1.56)	−2.1 (1.52)	−1.7 (1.60)	<0.001 *
**Total Sleep Time**	**Overall**	**GAD < 10**	**GAD ≥ 10**	***p***
Short Sleep	477 (47.4%)	286 (41.1%)	191 (61.4%)	<0.001 *
Recommended Sleep	480 (47.7%)	380 (54.6%)	100 (32.2%)	
Long Sleep	50 (5.0%)	30 (4.3%)	20 (6.4%)	
**Insomnia Category**	**Overall**	**GAD < 10**	**GAD ≥ 10**	***p***
No Insomnia	350 (34.8%)	315 (45.3%)	35 (11.3%)	<0.001 *
Subthreshold Insomnia	389 (38.6%)	270 (38.8%)	119 (38.3%)	
Moderate Insomnia	212 (21.1%)	97 (13.9%)	115 (37.0%)	
Severe Insomnia	56 (5.6%)	14 (2.0%)	42 (13.5%)	

Mean (SD); *n* (%); GAD: Generalized Anxiety Disorder; MAP: Multivariable Apnea Prediction Score; * *p* < 0.05 is considered statistically significant; Income quintile 1, lowest socioeconomic status; Income quintile 5, highest socioeconomic status.

**Table 2:** Insomnia components and severity of anxiety in the whole sample and among short sleepers.

Model	1: Unadjusted			2: Adjusted ^†^			3: Short Sleepers ^†^		
Insomnia Component	β	95% CI	*p*	β	95% Cl	*p*	β	95% CI	*p*
Nighttime Disturbance	0.88	0.44, 1.3	<0.001 *	0.87	0.43, 1.3	<0.001 *	0.69	0.03, 1.4	0.042 *
Daytime Dysfunction	1.30	0.81, 1.8	<0.001 *	1.20	0.76, 1.7	<0.001 *	1.20	0.55, 1.9	<0.001 *
Self-Perceived Dissatisfaction	1.20	0.60, 1.7	<0.001 *	1.10	0.54, 1.6	<0.001 *	0.92	0.10, 1.7	0.027 *

^†^ Adjusted covariates: age, sex, race/ethnicity, education, income quintile, MAP score, body mass index; * *p* < 0.05 is considered statistically significant.

**Table 3:** The associations between insomnia components and symptoms of anxiety.

Model	1: Unadjusted			2: Adjusted ^†^			3: Short Sleepers ^†^		
Anxious Feelings	OR	95% CI	*p*	OR	95% CI	*p*	OR	95% Cl	*p*
Nighttime Disturbance	1.15	0.93, 1.40	0.2000	1.22	0.98, 1.51	0.0720	1.26	0.94, 1.70	0.1300
Daytime Dysfunction	1.73	1.39, 2.16	<0.001 *	1.71	1.36, 2.16	<0.001 *	1.66	1.21, 2.29	0.002 *
Self-Perceived Dissatisfaction	1.30	1.01, 1.69	0.0420 *	1.30	1.00, 1.70	0.0540	1.17	0.81, 1.70	0.4000
**Inability to Control Worrying**	**OR**	**95% CI**	***p***	**OR**	**95% CI**	***p***	**OR**	**95% Cl**	***p***
Nighttime Disturbance	1.21	0.99, 1.49	0.0640	1.27	1.02, 1.57	0.0290 *	1.37	1.03, 1.84	0.0330 *
Daytime Dysfunction	1.55	1.24, 1.93	<0.001 *	1.54	1.23, 1.94	<0.001 *	1.40	1.04, 1.91	0.0270 *
Self-Perceived Dissatisfaction	1.54	1.18, 2.00	0.0010 *	1.50	1.14, 1.96	0.0030 *	1.30	0.90, 1.86	0.2000
**Excess Worrying**	**OR**	**95% CI**	***p***	**OR**	**95% CI**	***p***	**OR**	**95% Cl**	***p***
Nighttime Disturbance	1.23	1.00, 1.51	0.0460 *	1.29	1.04, 1.59	0.0190 *	1.33	1.00, 1.78	0.0530
Daytime Dysfunction	1.62	1.31, 2.03	<0.001 *	1.61	1.28, 2.02	<0.001 *	1.56	1.16, 2.12	0.0040 *
Self-Perceived Dissatisfaction	1.57	1.22, 2.04	<0.001 *	1.57	1.20, 2.05	<0.001 *	1.27	0.89, 1.82	0.2000
**Trouble Relaxing**	**OR**	**95% CI**	***p***	**OR**	**95% CI**	***p***	**OR**	**95% Cl**	***p***
Nighttime Disturbance	1.17	0.95, 1.44	0.1400	1.18	0.95, 1.46	0.1400	1.05	0.79, 1.41	0.7000
Daytime Dysfunction	1.42	1.13, 1.77	0.0020 *	1.39	1.11, 1.75	0.0050 *	1.43	1.06, 1.93	0.0200 *
Self-Perceived Dissatisfaction	1.89	1.45, 2.47	<0.001 *	1.92	1.46, 2.53	<0.001 *	1.77	1.24, 2.56	0.0020 *
**Restlessness**	**OR**	**95% CI**	***p***	**OR**	**95% CI**	***p***	**OR**	**95% Cl**	***p***
Nighttime Disturbance	1.57	1.22, 2.03	<0.001 *	1.39	1.08, 1.82	0.0130 *	1.37	0.97, 1.93	0.0730
Daytime Dysfunction	1.63	1.25, 2.15	<0.001 *	1.65	1.24, 2.20	<0.001 *	1.69	1.19, 2.45	0.0040 *
Self-Perceived Dissatisfaction	1.25	0.90, 1.74	0.2000	1.25	0.88, 1.76	0.2000	1.22	0.79, 1.89	0.4000
**Irritability**	**OR**	**95% CI**	***p***	**OR**	**95% CI**	***p***	**OR**	**95% Cl**	***p***
Nighttime Disturbance	1.28	1.04, 1.57	0.0210 *	1.25	1.01, 1.54	0.0430 *	1.21	0.91, 1.62	0.2000
Daytime Dysfunction	1.59	1.27, 1.98	<0.001 *	1.55	1.23, 1.95	<0.001 *	1.59	1.18, 2.17	0.0030 *
Self-Perceived Dissatisfaction	1.35	1.04, 1.76	0.0240 *	1.36	1.04, 1.78	0.0260 *	1.27	0.88, 1.82	0.2000
**Catastrophizing**	**OR**	**95% CI**	***p***	**OR**	**95% CI**	***p***	**OR**	**95% Cl**	***p***
Nighttime Disturbance	1.31	1.04, 1.65	0.0220 *	1.26	1.00, 1.61	0.056	1.19	0.87, 1.64	0.3000
Daytime Dysfunction	1.25	0.98, 1.61	0.0710	1.20	0.93, 1.55	0.2000	1.28	0.92, 1.79	0.1500
Self-Perceived Dissatisfaction	1.52	1.13, 2.05	0.0060 *	1.52	1.12, 2.07	0.0080 *	1.16	0.78, 1.74	0.5000

^†^ Adjusted covariates: age, sex, race/ethnicity, education, income quintile, MAP score and body mass index; * *p* < 0.05 is considered statistically significant.

**Table 4:** Forward Stepwise Regression Analysis of Insomnia Components Associated with Each Anxiety Symptom, Adjusted for Sociodemographics.

		Rank	OR	95% CI	*p*
Anxious Feelings	Nighttime	N/A			
	Daytime	1	1.31	(1.18, 1.45)	<0.001 *
	Self-Perceived Dissatisfaction	2	1.21	(1.09, 1.34)	<0.001*
Inability to Control Worrying	Nighttime	3	1.09	(1.01, 1.18)	0.032 *
	Daytime	1	1.23	(1.11, 1.37)	<0.001 *
	Self-Perceived Dissatisfaction	2	1.22	(1.08, 1.37)	0.001 *
Excess Worrying	Nighttime	3	1.10	(1.02, 1.19)	0.017 *
	Daytime	1	1.26	(1.14, 1.40)	<0.001 *
	Self-Perceived Dissatisfaction	2	1.23	(1.10, 1.39)	0.001 *
Trouble Relaxing	Nighttime	N/A			
	Daytime	2	1.17	(1.06, 1.30)	0.003 *
	Self-Perceived Dissatisfaction	1	1.43	(1.27, 1.58)	<0.001 *
Restlessness	Nighttime	2	1.18	(1.09, 1.28)	<0.001 *
	Daytime	1	1.33	(1.19, 1.48)	<0.001 *
	Self-Perceived Dissatisfaction	N/A			
Irritability	Nighttime	3	1.08	(1.00, 1.17)	0.042 *
	Daytime	1	1.23	(1.11, 1.36)	<0.001 *
	Self-Perceived Dissatisfaction	2	1.16	(1.03, 1.31)	0.018 *
Catastrophizing	Nighttime	N/A			
	Daytime	N/A			
	Self-Perceived Dissatisfaction	1	1.43	(1.31, 1.55)	<0.001 *

* *p* < 0.05 is considered statistically significant; adjusted covariates: age, sex, race/ethnicity, education, and body mass index.

## Data Availability

Not applicable.
